# Band Structure Engineering and Thermoelectric Properties of Charge-Compensated Filled Skutterudites

**DOI:** 10.1038/srep14641

**Published:** 2015-10-12

**Authors:** Xiaoya Shi, Jiong Yang, Lijun Wu, James R. Salvador, Cheng Zhang, William L. Villaire, Daad Haddad, Jihui Yang, Yimei Zhu, Qiang Li

**Affiliations:** 1 Condensed Matter Physics and Materials Science Department, Brookhaven National Laboratory, Upton, New York 11973, USA; 2 Materials Science & Engineering Department, University of Washington, Seattle, WA 98195, USA; 3 Chemical and Materials Systems Lab, General Motors R&D Center, Warren, MI 48090, USA; 4 Fuel Systems Engineering, General Motor Global Design, Engineering and Product Programs, Warren, MI 48090-9020.

## Abstract

Thermoelectric properties of semiconductors are intimately related to their electronic band structure, which can be engineered via chemical doping. Dopant Ga in the cage-structured skutterudite Co_4_Sb_12_ substitutes Sb sites while occupying the void sites. Combining quantitative scanning transmission electron microscopy and first-principles calculations, we show that Ga dual-site occupancy breaks the symmetry of the Sb-Sb network, splits the deep triply-degenerate conduction bands, and drives them downward to the band edge. The charge-compensating nature of the dual occupancy Ga increases overall filling fraction limit. By imparting this unique band structure feature, and judiciously doping the materials by increasing the Yb content, we promote the Fermi level to a point where carriers are in energetic proximity to these features. Increased participation of these heavier bands in electronic transport leads to increased thermopower and effective mass. Further, the localized distortion from Ga/Sb substitution enhances the phonon scattering to reduce the thermal conductivity effectively.

Filled skutterudites have been widely studied for decades due to their high thermoelectric performance^1^,^2^,^3^,^4^,^5^,^6^,^7^,^8^,^9^,^10^, which is characterized by the dimensionless figure of merit, *zT* = *S*^*2*^*T*/*ρ∙κ*, where *S* is the thermopower, *ρ* is the electrical resistivity, *T* is the absolute temperature, and *κ* is the total thermal conductivity (*κ* *= κ*_*e*_ *+* *κ*_*L*_ where *κ*_*e*_ and *κ*_*L*_ are the electronic and lattice contributions to the total thermal conductivity, respectively). The intrinsic caged structure of the skutterudites enables different guest atoms, such as alkali^11^,^12^,^13^, alkaline earth^14^,^15^,^16^, and lanthanide metals^6^,^7^,^17^,^18^,^19^, to fill these voids. The fillers play dual roles in the skutterudites by tuning the electrical performance as well as reducing *κ*_*L*_. Take n-type filled skutterudites as an example, the electrical performance, or power factor (*S*^2^/*ρ*) can be optimized by adjusting the filling fractions and as a result the electron carrier concentrations. Simultaneously, *κ*_*L*_ can be greatly suppressed by the rattling modes of the fillers^20^,^21^.

Recent studies on the group IIIA doped Co_4_Sb_12_^22^,^23^,^24^, particularly Ga, suggest another strategy to further reduce thermal conductivity by introducing so called “charge-compensated compound defect (CCCD)”^25^,^26^. CCCD does not change the carrier concentration of the compound due to the dual-site occupancy nature of Ga impurities in the Co_4_Sb_12_ lattice. It has been established from first-principles calculation, transport properties, microstructural and compositional variation studies that Ga has the ability to occupy both 2*a*-voids and 24*g*-Sb Wyckoff sites^26^. This dual-site occupancy produces effective scattering of both the low and high-frequency lattice phonons, hence further reduction in the lattice thermal conductivity. The drawback of this strategy is the Ga-CCCD alone provides limited tuning range of the carrier concentration that it may fall outside what is needed to achieve the optimal electrical performance in skutterudites. To significantly increase the carrier concentration of Ga-doped skutterudites, electron or hole donors, for example a second filler in n-type skutterudites, are required. Then, the question is how their structural and thermoelectric properties respond to the introduction of a second “rattling” filler and change in carrier concentration. This question cannot be satisfactorily answered without the understanding of both their crystal and electronic structure, which is currently lacking.

Here, we report the synthesis, microstructure and thermoelectric properties of Ga and Yb-containing charge-compensated compounds, in conjunction with the first-principles calculation of their electronic band structures. The calculation shows that Ga dual-site occupancy breaks the symmetry of the Sb-Sb network. As a result, the triply-degenerate conduction bands, which are normally too deep to contribute to charge transport are “softened” and split into several non-degenerate bands with lower energy that are closer to the conduction band edge. By introducing Yb atoms as second fillers (dopants), we were able to change the carrier concentration substantially and move the Fermi level up to meet the deep heavy conduction band. Synergistically, the charge-compensating nature of the dual occupancy Ga increases the filling fraction limit of the second filler atoms, the Yb in this study. Through a quantitative Scanning Transmission Electron Microscopy (STEM) investigation we show, for the first time, direct evidence for the distorted skutterudite cages as well as the off-center-cage filling behavior imparted by Ga-CCCD doping. We find that Ga’s charge-compensating nature can coexist with other filler atoms, specifically Yb, and that it is feasible to tune the thermoelectric performance to the optimal level without losing the benefit from CCCD. Significantly enhanced thermopower was achieved, particularly in the samples with high carrier concentrations, by only varying Yb filling fractions in the Ga-CCCD skutterudites, as the result of merging the Fermi level with the deeper and heavier electron bands. Electron Probe Micro-Analysis (EPMA) of these samples show much higher Yb solubility as compared to Yb-filled skutterudites lacking Ga. Combining higher thermopower and lower lattice thermal conductivity, *zT* values in Ga charge-compensated skutterudites were enhanced through this band structure engineering.

## Results

### Lattice parameters and filling fractions

**Figure 1:**
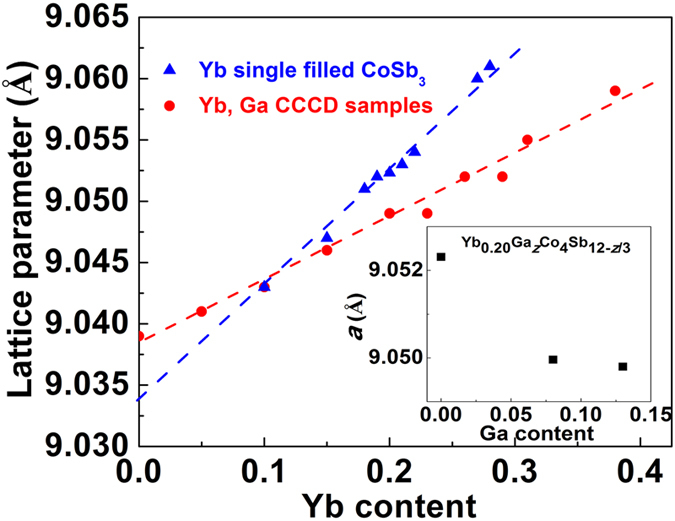
Lattice parameter (*a*) as a function of Yb content. Blue triangles are Yb single filled Co_4_Sb_12_ and red circles represent Yb, Ga CCCD samples: Yb_*x*_Ga_0.2_Co_4_Sb_11.9333_ (*x *= 0, 0.05, 0.10, 0.15, 0.20, 0.26) and Yb_*y*_Ga_0.15_Co_4_Sb_11.95_ (*y *= 0.25, 0.30, 0.35, 0.40). The data of Yb single filled Co_4_Sb_12_ are from this study and taken from the literatures^4^,^6^,^7^. The dashed lines are the trend for eye guidance. The inserted figure plots the lattice parameters of Yb_0.20_Ga_*z*_Co_4_Sb_12-*z*/3_ (*z *= 0, 0.10, 0.15) as a function of Ga content.

Figure 1 shows the lattice parameters (***a***) of the samples as a function of the Yb content determined by EPMA. The lattice parameters of this study were calculated from powder XRD data by Rietveld refinement. The detailed experimental data of lattice parameters and actual compositions can be found in supporting information - Supplementary Table 1 and Supplementary Figure 1. The lattice parameter of Ga_0.2_Co_4_Sb_11.9333_ sample is 9.039 Å, which is larger than that of unfilled skutterudites Co_4_Sb_12_ (~9.034 Å)^3^. The expansion of the lattice is due to partial Ga filling at the 2*a* site, consistent with the previous findings^25^. The lattice parameter of Yb, Ga-CCCD samples increases monotonically with increasing Yb content up to 38% of 2*a* site occupancy. It is important to notice that this filling fraction of Yb is much higher than the filling fraction limit from the previous experiment and the theoretical prediction on the Yb single filled Co_4_Sb_12_, which is around 22% of the cage filling fraction^27^,^28^. According to our EPMA data, the actual Yb chemical composition in the nominal Yb_0.40_Ga_0.15_Co_4_Sb_11.95_ reaches as high as 0.38 without any tendency of saturation. We believe that the Ga-CCCD is responsible for the enhancement of filling fraction of Yb in the Co_4_Sb_12_. Yb acts as the second filler into the Ga charge-compensated matrix, and competes with filler site Ga thermodynamically. As such, the filler site Ga has a tendency to move partially to Sb site. The introduction of more electron deficient Ga onto Sb site boosts the ability of the compound to absorb more donated free charges from the second filler atom, Yb in this case, and hence increases carrier concentration and the filling fraction limit of Yb. A recent theoretical study indeed shows that the filler site Ga tends to partially move to Sb site at higher carrier concentrations^29^. Structurally, a lattice contraction would be expected if the Ga does move to the Sb site. As demonstrated in Fig. 1, the lattice parameter of Yb, Ga-CCCD Co_4_Sb_12_ is smaller than that of Yb single filled Co_4_Sb_12_ under the high Yb filling fraction, indicating a contracted lattice due to the Ga-CCCD. This finding is consistent with the systematic lattice contraction observed in Ga_x_Yb_0.20_Co_4_Sb_12-*x*/3_ when varying the Ga content, shown in the inset of Fig. 1. The contraction of the lattice with assumed increasing Ga content on the Sb site reduces the lattice strain caused by the expansion imparted by increasing Yb content and is another reason for the increased solubility of Yb.

### STEM structural characterizations

**Figure 2:**
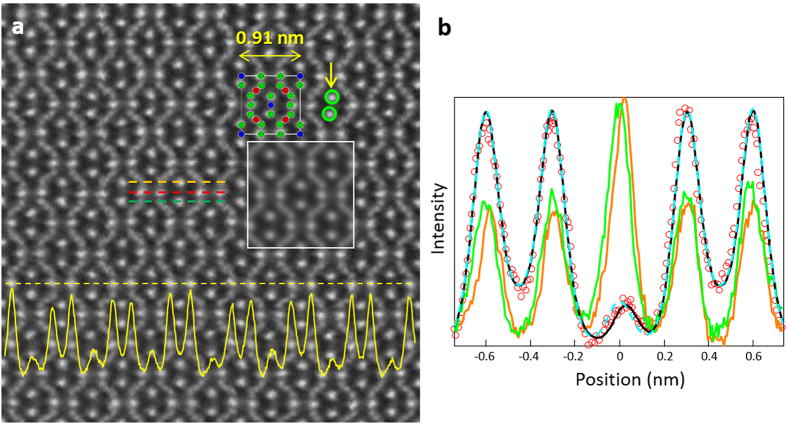
STEM analysis and structural modeling. (**a**) STEM-HAADF image (convergent angle: 21 mrad, collection angle: 67–275 mrad). The structure model (red, green and blue spheres represent Co, Sb and Ga/Yb atoms, respectively) and calculated image (inset with white outline) are embedded in the image. A line scan intensity profile from the yellow dashed line is shown at the bottom. The green circles pointed by the yellow arrow illustrate the misalignment of the Sb atoms, indicating the distortion of the Sb cage that is associated with the off-center-cage filling behavior of the filler atoms. (**b**) Intensity line profiles from the experimental image and simulations. The red circles are from the red scan line shown in (**a**), the light blue and black lines are from the calculations based on the structure models of Yb_0.26_Ga_0.2_Co_4_Sb_11.9333_ with filled Ga/Yb at center (light blue) and deviated 0.027 nm away from the center (black), respectively. The orange and green lines are from the orange and green scan lines in (**a**).

In order to directly determine the structural changes imparted by introducing Ga-CCCD into the skutterudite structure, high-resolution STEM images of Yb_0.26_Ga_0.2_Co_4_Sb_11.9333_ were taken with a double aberration-corrected transmission electron microscope. Figure 2 shows a high-angle annular dark-field (HAADF) STEM image viewed along the [001] direction, where the frame elements (Co and Sb) as well as the fillers are clearly distinguishable, as shown in the structure model highlighted in Fig. 2a. The cage distortion combined with the off-center-cage filling behavior is identified by comparing the experimental line scan profiles with the simulation results, shown in Fig. 2b. The orange and green lines in Fig. 2b are from the orange and green scan lines in Fig. 2a which pass through the positions of Sb in the cage frame. In an ideal filled skutterudite, the two lines should overlap based on the structural symmetry. However, as shown in Fig. 2b, the peak positions shift between the orange and green lines, indicating the distortion of Sb atoms and the cage structure. As a matter of fact, we can clearly identify the distortion of the Sb cage as the atoms, indicated by two circles pointed by the yellow arrow in Fig. 2a, are not aligned vertically. Further, it is interesting to point out that the cage distortion is always associated with the off-center-cage filling behavior of the filler atoms. An ideal cage-center filling behavior, as seen in most filled skutterudites^10^, should produce perfectly symmetric double valleys in the profile intensity represented by the light blue simulated line scan shown in Fig. 2b. However, the experimental line scan profile (red dots in Fig. 2b) deviates from the light blue line (center-filling model) near the center filler position (0 nm position) relative to the 2*a* site in Fig. 2b. Instead, an off-center filling model, which is represented by the black line in Fig. 2b, fits the experimental results quite well. This off-center-cage feature was the result of a covalent bond between filler Ga and the host Sb cage that was partially substituted by Ga, in agreement with the first-principles calculations in ref. 26 and ref. 29. Here, STEM crystallographic data provides the first direct experimental evidence for the off-center-cage filling behavior of Ga filler atoms, that can be clearly identified in the longer range intensity line scan at the bottom of Fig. 2a. The variation of the image intensity of these Sb columns that indicates the partial replacement of Sb with Ga in Sb site is rather significant. Otherwise, the image intensity of the Sb columns in the line scan should be the same if they are fully occupied by Sb atoms, based on the crystal symmetry. The covalent bonding of Ga should be effective for scattering heat carrying phonons to further reduce the thermal conductivity of the material, which will be studied in the future.

### Electronic band structure

**Figure 3:**
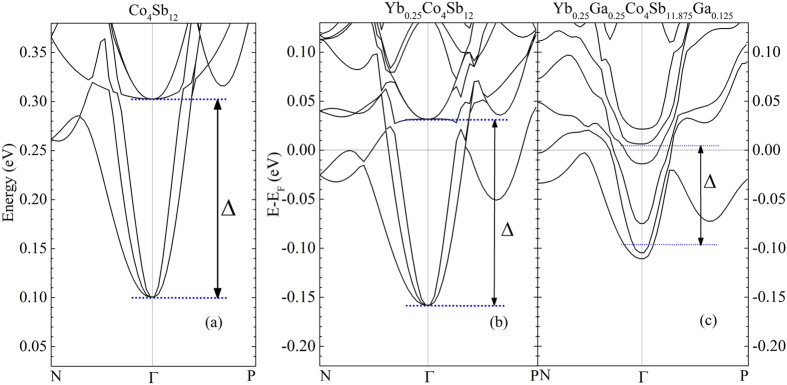
Theoretical calculation of electronic band structures. First-principles band structure calculations for (**a**) Co_4_Sb_12_, (**b**) Yb_0.25_Co_4_Sb_12_, and (**c**) Yb_0.25_Ga_0.25_Co_4_Sb_11.875_Ga_0.125_. The zero energy points in (**b**,**c**) represent the Fermi levels (E_F_s) of the two compounds.

First-principles band structure calculations have been carried out to shed light on the band variations caused by the introduction of Yb and Ga. Figure 3a shows the band structure of Co_4_Sb_12_ around its conduction band bottom (CBB). The CBB is located at the Γ point, and is composed of three degenerated bands. The next bands at the Γ point, ~0.2 eV above the CBB (denoted as Δ in Fig. 3a), are also triply-degenerated. Figure 3b shows the band structure of Yb_0.25_Co_4_Sb_12_ with zero energy point representing the Fermi level (E_F_) of the system. The conduction band of Yb_0.25_Co_4_Sb_12_ is similar to that of Co_4_Sb_12_, especially at the Γ point, the Δ is barely changed by the Yb filling (~0.19 eV), and the upper triple-degenerated bands are 0.03 eV higher than the E_F_, limiting their contribution to the electrical transport. The values of Δ are almost identical in all the n-type Co_4_Sb_12_-based skutterudites with alkali metal (Δ = 0.20 eV in K_0.25_Co_4_Sb_12_), alkali earth (Δ = 0.20 eV in Ba_0.25_Co_4_Sb_12_), or rare earth fillers, indicating that all the metallic fillers have negligible influence on the conduction band of Co_4_Sb_12_^30^.

The band structure changes remarkably with IIIA elements, as shown in Fig. 3c for Co_4_Sb_12_ with both Yb filling and Ga-CCCD, in which the ratio of Ga at the filler sites and the Sb sites is 2:1. The degeneracy of the CBB is broken by the Ga at the Sb sites. The most distinct variation relating with the electrical transport is the upper three degenerated bands split and shift downwards. The Δ, calculated from the averaged values of the upper three bands and those at CBB, decreases to 0.10 eV. The E_F_ (zero energy point) therefore crosses these upper bands and enhances the density of states around the E_F_, which is beneficial to the thermopower. We also calculated the band structure of Ga_0.25_Co_4_Sb_12_ and In_0.25_Co_4_Sb_12_ to justify the origin of the effect as shown in Supplementary Figure 2. In these calculations we assume the IIIA atoms are all at filler sites. The Δ values are 0.11 eV and 0.12 eV for the Ga- and In-filled, respectively. This result indicates that the downwards shifting of the upper three bands is caused by the filler Ga rather than the Ga substituting at the 24 *g* site. The change in the energy positions of bands in Co_4_Sb_12_ with Ga or In incorporation onto the 2*a* site is probably due to their differing filler-host bonding interactions. In contrast to more electropositive fillers, IIIA elements form polar-covalent bonds with the Co_4_Sb_12_ host, according to our first-principles study as well as ref. 29. The covalent character of the bond causes the off-center filling shown in the STEM results, and energetically lowers some of the anti-bonding bands of the host. In the perspective of transport, these shifted bands can contribute to the electronic states around E_F_s.

### Electrical transport properties

**Figure 4:**
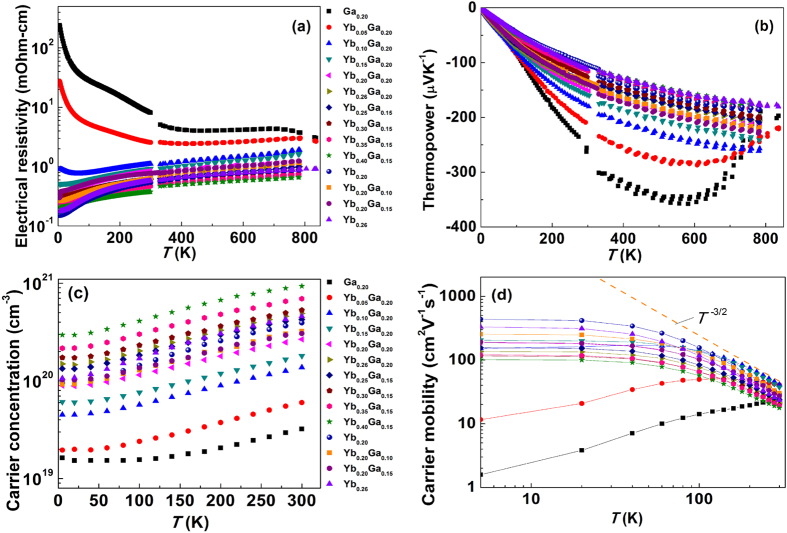
Electrical properties as a function of temperature. (**a**) Electrical resistivity, (**b**) thermopower, (**c**) carrier concentration and (**d**) carrier mobility of Yb_*x*_Ga_0.2_Co_4_Sb_11.9333_ (*x *= 0, 0.05, 0.10, 0.15, 0.20, 0.26), Yb_*y*_Ga_0.15_Co_4_Sb_11.95_ (*y *= 0.25, 0.30, 0.35, 0.40), Yb_0.20_Ga_*z*_Co_4_Sb_12-*z*/3_ (*z *= 0, 0.10, 0.15) and Yb_0.26_Co_4_Sb_12._ The dashed line in (**d**) represents the temperature dependence of *T*^−3/2^, indicating an acoustic phonon scattering trend line for eye guidance.

For simplicity of presentation, we label our specimens based on their nominal Yb and Ga content in the form of Yb_*A*_Ga_*B*_ for Ga-CCCD samples and Yb_*C*_ for Yb single filled Co_4_Sb_12_ samples. For example, the sample Yb_0.26_Ga_0.20_Co_4_Sb_11.9333_ is written as Yb_0.26_Ga_0.20_, while the sample Yb_0.20_Co_4_Sb_12_ is written as Yb_0.20._ The same rule will be applied in all the figures discussed in this section. Figure 4a shows electrical resistivity (*ρ*) of all the samples as a function of temperature. A transition from semiconducting behavior to metallic behavior is observed with increasing Yb content. Due to the charge-compensation between the void-filling Ga (nominally Ga^1+^) and Sb-substitutional Ga (nominally Ga^2−^), the Ga_0.20_ sample exhibits intrinsic semiconducting behavior and has the lowest carrier concentration (*n*), as shown in Fig. 4c. This behavior agrees quite well with the result reported previously and indicates invariance of this result with different sample preparation methods^26^. The electrical resistivity decreases with increasing Yb content, mainly due to the increased filling fraction of Yb and a concomitant increase in carrier concentration. Correspondingly, the absolute value of thermopower decreases with increasing Yb content. It is important to point out that Yb_0.26_ sample has a similar carrier concentration to that of Yb_0.26_Ga_0.20_ sample, as shown in Fig. 4c. However, Yb_0.26_Ga_0.20_ sample exhibits a significantly higher absolute thermopower value than that of Yb_0.26_ sample at all temperatures (Fig. 4b). Thermopower is related to the charge carrier density of states near Fermi level as expressed by the Mott relation^31^:


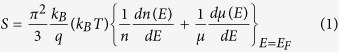


Here, *k*_*B*_is the Boltzmann’s constant, *q* the electronic charge, and *μ*(*E*) the carrier mobility. 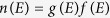 is the carrier density at the energy level *E*, where *g*(*E*) is the charge density of states and *f*(*E*) is the Fermi function. *E*_*F*_ is the Fermi level. As shown in Eq. 1, thermopower *S* is proportional to the energy dependence of charge density-*n*(*E*), which is determined by the electronic band structure of the materials. The enhanced thermopower of Yb_0.26_Ga_0.20_ as compared to Yb_0.26_ sample is attributed to the change of electronic band structure that we have shown in Fig. 3. The heavy bands, which are shifted down in energy by the introduction of Ga-CCCD, start to contribute to the density of states and thermopower in the high carrier concentration region.

The carrier concentration is tunable in these Ga-containing charge-compensated compounds in the same way that carrier concentration can be modified by controlling the filling fraction in Ga-free material, as shown in Fig. 4c. The Ga_0.20_ sample has the lowest carrier concentration of ~3 × 10^19^ cm^−3^ at 300 K, while Yb_0.40_Ga_0.15_ sample has the highest carrier concentration level ~9 × 10^20^ cm^−3^ at 300 K in this study. We conclude, based on the monotonic increase in both the lattice parameters and carrier concentration with increasing Yb content, that we can reach a filling fraction of 0.38 for Yb. This is higher than the filling fraction limit calculated for Yb_*x*_Co_4_Sb_12_ and is believed to be the result of the charge-compensating nature of the Ga^28^,^29^.

We have also calculated the low temperature carrier mobility (*μ*_*H*_) of all the samples and plotted their temperature dependence in Fig. 4d. For typical n-type filled skutterudites with moderate to high *n*, acoustic phonon scattering dominates near room temperature^32^, leading to a 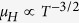, as the trend line highlights in Fig. 4d. Ga_0.20_ and Yb_0.05_Ga_0.20_ samples show positive temperature dependence of carrier mobility, indicating the presence of more than one dominant scattering mechanism, most likely a mixture of acoustical phonon and ionized impurity scattering (a purely ionized impurity scattering has a 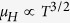
relation). In low carrier concentration samples, ionized impurity scattering makes the dominant contribution to the transport, while acoustic phonon scattering becomes dominant when increasing carrier concentrations. The Ga-CCCD is responsible for the ionized impurity scattering as the Sb-substitutional Ga introduces ionized impurity centers, which greatly influence the carrier mobility, especially at low temperatures. It is known that the Sb-rings provide conduction paths in the skutterudites, and substitution of Sb with Ga will distort the conduction framework and hence affect the electrical transport properties greatly.

### Thermal transport properties

**Figure 5:**
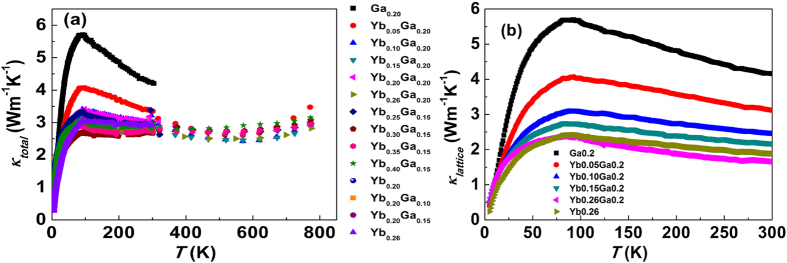
Thermal conductivity as a function of temperature. (**a**) Total thermal conductivity of all the samples and (**b**) lattice thermal conductivity of Yb_*x*_Ga_0.2_Co_4_Sb_11.9333_ (*x* = 0, 0.05, 0.10, 0.15, 0.26) and Yb_0.26_Co_4_Sb_12_.

The temperature dependence of the total thermal conductivity of all the samples is plotted in Fig. 5a. In order to show clearly the effect of Ga-CCCD to the thermal conductivity, only the low temperature (5 K–300 K) lattice thermal conductivity of Yb_*x*_Ga_0.2_Co_4_Sb_11.9333_ (*x *= 0, 0.05, 0.10, 0.15, 0.26) is shown in Fig. 5b. And the data of Yb_0.26_ sample is also included for comparison. The lattice thermal conductivity is obtained by subtracting the electronic component (*κ*_*e*_) from total thermal conductivity, where *κ*_*e*_ is determined by Wiedemann-Franz law (*κ*_*e *_= *L*_*0*_*T*/*ρ*). *L*_*0*_is the Lorenz number. A constant Lorenz number *L*_*0 *_= 2.0 × 10^−8^ V^2^K^−2^ is applied to calculate all *κ*_*e*_ values. Besides benefitting the electrical performance by improving thermopowers, the introduction of Ga-CCCD into filled skutterudites also has positive effects by reducing the thermal conductivity of the materials. As shown in Fig. 5, both *κ* and *κ*_*L*_ of Ga_0.20_ are significantly reduced compared to binary Co_4_Sb_12_, with the values of 4.3 Wm^−1^K^−1^ at 300 K for Ga_0.2_ as compared to Co_4_Sb_12_ whose room temperature value is 10 Wm^−1^K^−1^ 1. The reduction in *κ* could be explained by the dual-character phonon scattering mechanism proposed previously^25^, in that both resonant scattering from void-filled Ga and point defect scattering from Sb-substitutional Ga combine to more strongly reduce *κ*_*L*_ compared to void filling alone. We found a lower *κ*_*L*_ for Yb_0.26_Ga_0.20_ sample as compared to that of Yb_0.26_ in Fig. 5b, further supporting the role of Ga plays in reducing *κ* and *κ*_*L*_. The detailed study of thermal conductivity will be reported elsewhere. Suffice it to say that we conclude that the *κ*_*L*_ reduction is attributed to both the point defects from Ga/Sb substitution as well as a different localized vibration frequency as fillers from Ga in addition to Yb filled skutterudites.

### Thermoelectric figure of merit *zT*

**Figure 6:**
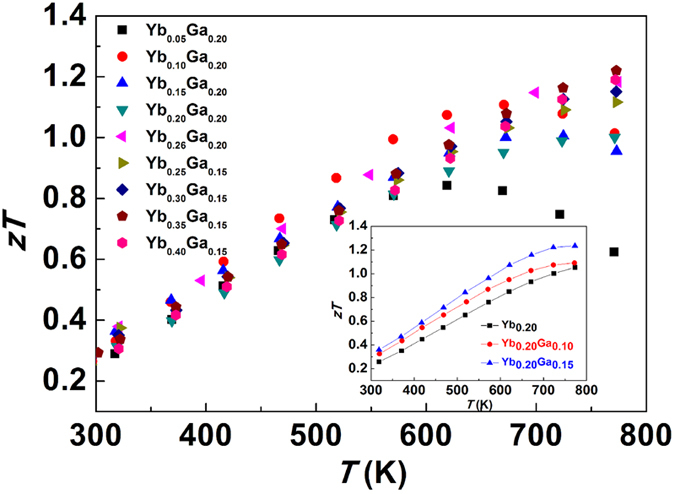
Thermoelectric figure of merit *zT* as a function of temperature for all the samples. Temperature dependence of *zT* of Yb_0.20_Ga_*z*_Co_4_Sb_12-*z*/3_ (*z* = 0, 0.10, 0.15) was plotted in the inserted figure separately to show the influence from Ga-CCCD to the thermoelectric performance.

Figure 6 shows the calculated *zT* values for all the Yb, Ga-CCCD skutterudites. The *zT* value is enhanced at higher Yb content and the highest *zT* reaches 1.25 at 780 K in the sample Yb_0.35_Ga_0.15_, comparable to the best performing Yb single filled skutterudites. The inset of Fig. 6 shows the *zT* value greatly enhanced by introducing Ga-CCCD with the same Yb filling fraction. The thermoelectric performance improvement is mainly attributed to its unique electronic band structure as well as mixed phonon scattering mechanism produced by Ga-CCCD. The thermoelectric performance of Ga-containing CCCD materials can be optimized by adjusting filling fraction of the secondary filler atoms, like Yb in this study.

## Discussion

**Figure 7:**
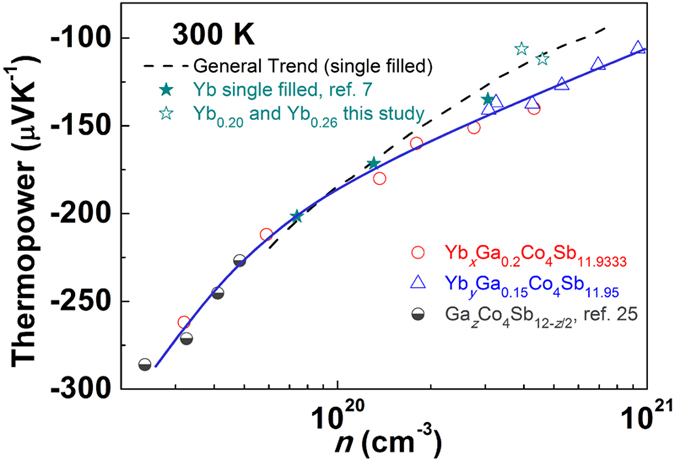
Thermopower values at different carrier concentration levels at 300 K. The dashed line represents the general trend of thermopower taken from the literature for n-type filled skutterudites with different single fillers^25^. The red circle symbols are Yb_*x*_Ga_0.2_Co_4_Sb_11.9333_ (*x* = 0, 0.05, 0.10, 0.15, 0.20, 0.26) and the blue triangles are Yb_*y*_Ga_0.15_Co_4_Sb_11.95_ (*y* = 0.20, 0.25, 0.30, 0.35, 0.40) from this study. The data of Ga-CCCD samples Ga_*δ*_Co_4_Sb_12-*δ*/3_ from ref. 25 is also plotted in the figure. The solid blue line represents the data trend for all the Ga-containing charge-compensated compounds.

The influence to the thermoelectric properties from electronic band structure change could be better understood by investigating thermopower at different carrier concentration. Figure 7 shows the carrier concentration dependence of thermopower for all samples at 300 K. The general trend line was drawn by fitting thermopower of n-type single filled-Co_4_Sb_12_ from literature data^25^. We highlighted the Yb single filled Co_4_Sb_12_ data from ref. 7 with the solid star symbols, and in order to distinguish our experimental result from the literature data, the thermopower values of single filled samples Yb_0.20_ and Yb_0.26_ from this study were plotted using hollow star symbols in the figure. The Yb single filled samples follow the general trend line relatively well. However, the Ga-containing samples behave very differently, especially for the samples with high carrier concentrations. A solid blue trend line was drawn based on the thermopower values of Yb, Ga-CCCD samples and that of the Ga-CCCD Co_4_Sb_12_ from ref. 25. A crossover between the Ga-free filled skutterudites trend line and the Ga-containing CCCD samples trend line is shown in Fig. 7.

At low carrier concentrations, the absolute values of thermopower of the Yb, Ga-CCCD samples are slightly smaller than the extrapolated values of Ga-free single filled skutterudites. By increasing carrier concentration above 1.0 × 0^20^ cm^−3^, the absolute thermopower value exceeds that of Ga-free single filled skutterudites. At high carrier concentrations (>3 × 10^20^ cm^−3^), the absolute thermopower value of Yb, Ga sample is nearly 20% larger than that of the normal single filled skutterudites at the same carrier concentration. This special thermopower trend of Ga-containing samples can be explained by the electronic band structure result shown in Fig. 3. Due to the band split and reduced degeneracy at the conduction band edge, our Ga-containing samples exhibit smaller absolute *S* value compared with that of Ga-free filled skutterudites at low carrier concentrations. By gradually increasing Yb content, the Fermi level is pushed deeper into the conduction bands and goes across the three bands near conduction band edge. Thus, the absolute *S* gradually increases to the value comparable to that of Ga-free filled skutterudites. By further increasing the Yb filling fraction, we were able to tune the Fermi level to merge with the heavier bands whose energy has been decreased. On the other hand, the Yb filling fraction limit is increased by introducing Ga-CCCD, enabling us to tune the carrier concentration/Fermi level to much higher range. The enhanced electronic density of states from these heavier bands, which are normally inaccessible in Ga-free filled skutterudites, contributes additionally to the absolute thermopower value at high carrier concentrations, resulting in significantly enhanced thermopower compared with that of Ga-free filled skutterudites.

The change of the effective mass as a function of carrier concentration can also be understood from the calculated band structures. Assuming acoustic phonon scattering and a single parabolic band, the effective mass is obtained from the measured thermopower and carrier concentration, by using the following equations:


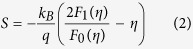



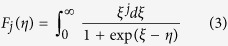



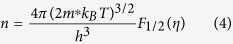


Here *h* is the Planck constant and 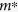 is the density of states effective mass. 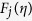 are the Fermi integrals of order *j*, 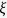 is the reduced carrier energy (*E/k*_*B*_*T*), and 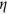 represents the reduced Fermi energy (*E*_*F*_*/k*_*B*_*T*). The value of 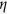 is obtained from experimental values of *S* from Equation 2. However, for skutterudites, a two-band Kane model, well-accepted for explaining the relation between *S*, *n* and 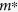 is applied here to understand the effective mass increase with the increase of *n* using the relationship^33^,^34^:


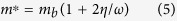


where 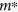 is the effective mass of the compound, *m*_*b*_ is the effective mass at the bottom of the band and 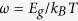 is the reduced band gap energy.

**Figure 8:**
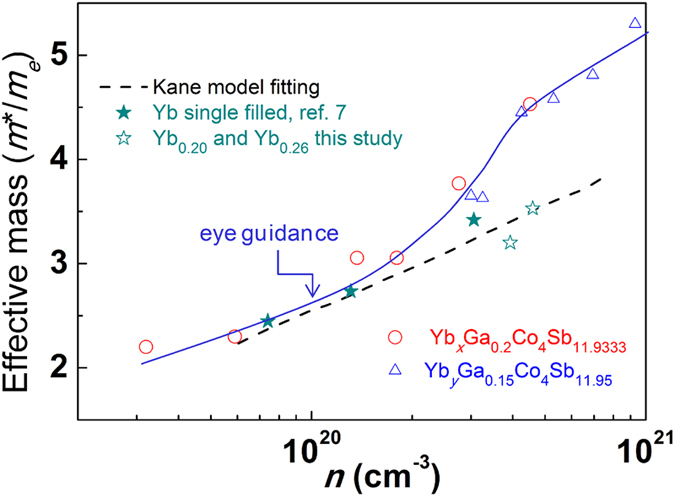
Carrier concentration dependence of effective mass at 300 K. The dashed line is a fit of the Yb_*y*_Co_4_Sb_12_ data using the Kane model with *m*_*b *_= 2.2 *m*_*e*_, and *E*_*g *_= 0.19 eV. The solid blue line represents the trend for eye guidance.

Figure 8 shows the carrier concentration dependence of the effective mass, where we applied the Kane model to the Yb single filled skutterudites data, from both our samples and the data from ref. 7, and obtained fitting parameters *m*_*b*_ = 2.2 *m*_*e*_ and *E*_*g *_= 0.19 eV. The fitting result for effective mass and the band gap agree with prior measurements and theoretical calculations^15^,^35^,^36^. As shown in Fig. 8, there is a substantial increase of effective mass in the Yb, Ga-CCCD samples with high level Yb filling fraction while the effective mass of low carrier concentration samples is quite similar as that of Yb single filled skutterudites. This is further experimental evidence to support the first-principles calculation results and shows the influence of the electronic band structure change on the transport properties introduced by Ga-CCCD.

In summary, our findings shed the light on the nature of the dual-site occupancy of Ga on the electronic band structure and the thermoelectric properties of the Yb, Ga filled skutterudites. The cage distortion and off-cage-center filling behavior has been directly observed through the analysis of atomic imaging. The carrier concentration of the charge-compensated skutterudites is tunable by adding the second filler. Ga/Sb substitution creates ionized impurity defects, resulting in a mixed electron scattering mechanisms in the materials. A distinct deviation of thermopower from the normal filled skutterudites trend, coupled with sharp increase of effective mass, has been observed in the Yb, Ga filled compounds with high carrier concentration. This is attributable to the electronic band structure change after introducing Ga-CCCD. We found the Yb, Ga-CCCD samples show lower thermal conductivity compared with that of Yb single filled samples with the same Yb filling fractions. The cage site Ga plays a role in providing additional vibration modes for the Yb filler atoms, combined with the Ga-substitutional point defects. The optimized high temperature *zT* reaches 1.25 at 780 K in the sample Yb_0.35_Ga_0.15_Co_4_Sb_11.95_. Our study points to the possibility of electronic band structure engineering and thermal conductivity engineering in the filled skutterudites by introducing Ga-containing or similar charge-compensated defects. The present study can be extended to a broad range of thermoelectric materials for optimizing their electrical and thermal properties simultaneously.

## Methods

### Sample preparation

High-purity elements Co (99.999%, shot), Sb (99.999%, shot), Yb (99.98% ingot), and Ga (99.999% ingot) were used as the starting materials and were weighted to achieve Yb_x_Ga_0.2_Co_4_Sb_11.9333_ (x = 0, 0.05, 0.10, 0.15, 0.20, 0.26), Yb_y_Ga_0.15_Co_4_Sb_11.95_ (y = 0, 0.25, 0.30, 0.35, 0.40) and Yb_0.20_Ga_*z*_Co_4_Sb_12-*z*/3_ (*z *= 0, 0.10, 0.15), respectively. The stoichiometric quantities of the elements were loaded into carbon-coated quartz tubes and sealed under vacuum. The sealed ampoules were induction heated and melted twice. The obtained ingots were put into quartz crucible with a 0.5 mm diameter nozzle, induction melted and ejected under a pressure of 0.02 MPa of argon gas onto the high-speed rotating copper wheel with the linear speed of 20 m/s. The melt-spun ribbons were sintered using both hot press and spark plasma sintering (SPS) methods. The hot press sintering was carried out at 620 °C under 50 MPa pressure while the SPS samples were sintered at 625 °C under a uniaxial load of 50 MPa. For both of the hot press and SPS processing the pressure and temperature were held for 20 min before allowing the sample to cool to room temperature naturally with the load removed. The density of all the samples was above 95% of the theoretical value. The resulting billets were cut into bars for transport property measurements. TEM specimens were prepared by mechanical slicing, polishing, and dimpling, and followed by ion-milling until electron transparency was achieved.

### Structural characterization

Identification of the crystalline phase was determined by powder X-ray diffraction (XRD) at room temperature, which was carried out on a Philips 3100E diffractometer (Cu K radiation, λ = 0.15418 nm, 40 kV/30 mA). The phase purity and quantitative elemental analysis of the sintered bulk samples were determined by using Electron Probe Micro-Analysis (EPMA) by averaging the measurements at eight randomly selected spots on polished sample surfaces. EPMA was performed using a Cameca SX100 Electron Probe Micro Analyzer at an accelerating voltage of 20 kV. The STEM-HAADF images were taken with double aberration-corrected JEOL-ARM200F with convergent angle of 21 mrad and collection angle from 67 to 275 mrad. The STEM image calculations were carried out with computer codes, written at BNL, running on Graphics Processing Unit (GPU) based on the multislice method with the frozen phonon approach.

### First-principles calculations

First principles calculations were performed with the Vienna *ab initio* simulation package (VASP)^37^. We adopt generalized gradient approximation (GGA) functional of Perdew-Burke-Ernzerhof (PBE) form and projected augmented wave (PAW) method^38^,^39^,^40^. In order to simulate the compositions close to our experiments, 2*2*2 supercells of the primitive unit Co_4_Sb_12_, which contains 128 framework atoms and 8 filler sites, were constructed and used throughout all the calculations. For skutterudites with fillers and/or CCCDs, several atomic configurations are tested, and the most stable configurations are adopted. The supercells are fully relaxed before band structure calculations.

### Thermoelectric transport properties measurements

The low-temperature (5 K to 300 K) thermoelectric property measurements (electrical resistivity, *ρ*, thermopower, *S*, thermal conductivity, *κ*, and Hall coefficient, *R*_*H*_) were carried out in the sintered samples with a Physical Property Measurement System (PPMS, Quantum Design). The Hall coefficients were measured by sweeping the magnetic field up to 5T in both positive and negative directions. The Hall carrier concentration, *n*, was calculated by using 1/*R*_*H*_*e*, where *e* is elementary charge. The Hall carrier mobility, *μ*_*H*_, was obtained according to the relation *μ*_*H *_= *R*_*H*_/*ρ*. The high temperature transport property measurements were carried out in the SPS samples. The thermopower and electrical resistivity was measured from 330 K to 770 K using a Linseis LSR-3 system. Thermal conductivity from room temperature to 770 K was assessed by measurement of the diffusivity, *λ* (Anter Flashline 3050 diffusivity analyzer), specific heat, *C*_*p*_ (Netzsch 404 c differential scanning calorimeter), and density, *ρ* (as assessed by mass and dimension of a polished SPS’d billet) of the samples using the equation *κ* = *λ*·*C*_*p*_*·ρ* and taking the product of these values as a function of temperature.

## Additional Information

**How to cite this article**: Shi, X. *et al.* Band Structure Engineering and Thermoelectric Properties of Charge-Compensated Filled Skutterudites. *Sci. Rep.* **5**, 14641; doi: 10.1038/srep14641 (2015).

## Supplementary Material

Supplementary Information
