# Supplementary material for: Band Structure Engineering and Thermoelectric Properties of Charge-Compensated Filled Skutterudites
Source: Sci Rep. 2015 Oct 12;5:14641. doi: 10.1038/srep14641 (PMC4600978; doi:10.1038/srep14641)
Supplement: Supplementary Information [file srep14641-s1.doc]

Supplementary information

**Band Structure Engineering and Thermoelectric Properties of Charge-Compensated Filled Skutterudites**

Xiaoya Shi^1^, Jiong Yang^2^, Lijun Wu^1, a^, James R. Salvador^3, b^, Cheng Zhang^1^, William L. Villaire^4^, Daad Haddad^3^, Jihui Yang^2^, Yimei Zhu^1^, and Qiang Li^1, c^

1 Condensed Matter Physics and Materials Science Department,

Brookhaven National Laboratory, Upton, New York 11973, USA

2 Materials Science & Engineering Department

University of Washington, Seattle, WA 98195, USA

3 Chemical and Materials Systems Lab

General Motors R&D Center, Warren, MI 48090, USA

4 Fuel Systems Engineering

General Motor Global Design, Engineering and Product Programs

Warren, MI 48090-9020

| Nominal Composition | EMPA Composition | Lattice parameter (Å) |
| --- | --- | --- |
| Ga_0.20_Co_4_Sb_11.9333_ |  | 9.0393 |
| Yb_0.05_ Ga_0.20_Co_4_Sb_11.9333_ |  | 9.0414 |
| Yb_0.10_ Ga_0.20_Co_4_Sb_11.9333_ |  | 9.0435 |
| Yb_0.15_ Ga_0.20_Co_4_Sb_11.9333_ |  | 9.0463 |
| Yb_0.20_ Ga_0.20_Co_4_Sb_11.9333_ |  | 9.0494 |
| Yb_0.26_ Ga_0.20_Co_4_Sb_11.9333_ | Yb_0.24_ Ga_0.15_Co_4_Sb_11.87_ | 9.0527 |
| Yb_0.20_ Co_4_Sb_12_ | Yb_0.18_ Co_4_Sb_11.71_ | 9.0523 |
| Yb_0.20_ Ga_0.10_Co_4_Sb_11.9666_ | Yb_0.18_ Ga_0.08_Co_4_Sb_11.63_ | 9.0499 |
| Yb_0.20_ Ga_0.15_Co_4_Sb_11.95_ | Yb_0.18_ Ga_0.13_Co_4_Sb_11.65_ | 9.0498 |
| Yb_0.25_ Ga_0.15_Co_4_Sb_11.95_ | Yb_0.23_ Ga_0.14_Co_4_Sb_11.50_ | 9.0493 |
| Yb_0.30_ Ga_0.15_Co_4_Sb_11.95_ | Yb_0.29_ Ga_0.14_Co_4_Sb_11.50_ | 9.0525 |
| Yb_0.35_ Ga_0.15_Co_4_Sb_11.95_ | Yb_0.31_ Ga_0.14_Co_4_Sb_11.53_ | 9.0553 |
| Yb_0.40_ Ga_0.15_Co_4_Sb_11.95_ | Yb_0.38_ Ga_0.14_Co_4_Sb_11.47_ | 9.0592 |

Supplementary Table 1. Nominal composition, EMPA composition and lattice parameters. Samples are Yb*_x_*Ga_0.2_Co_4_Sb_11.9333_ (*x* = 0, 0.05, 0.10, 0.15, 0.20, 0.26), Yb*_y_*Ga_0.15_Co_4_Sb_11.95_ (*y* = 0.25, 0.30, 0.35, 0.40) and Yb_0.20_Ga*_z_*Co_4_Sb_12-_*_z_*_/3_ (*z* = 0, 0.10, 0.15) at room temperature for all the samples.


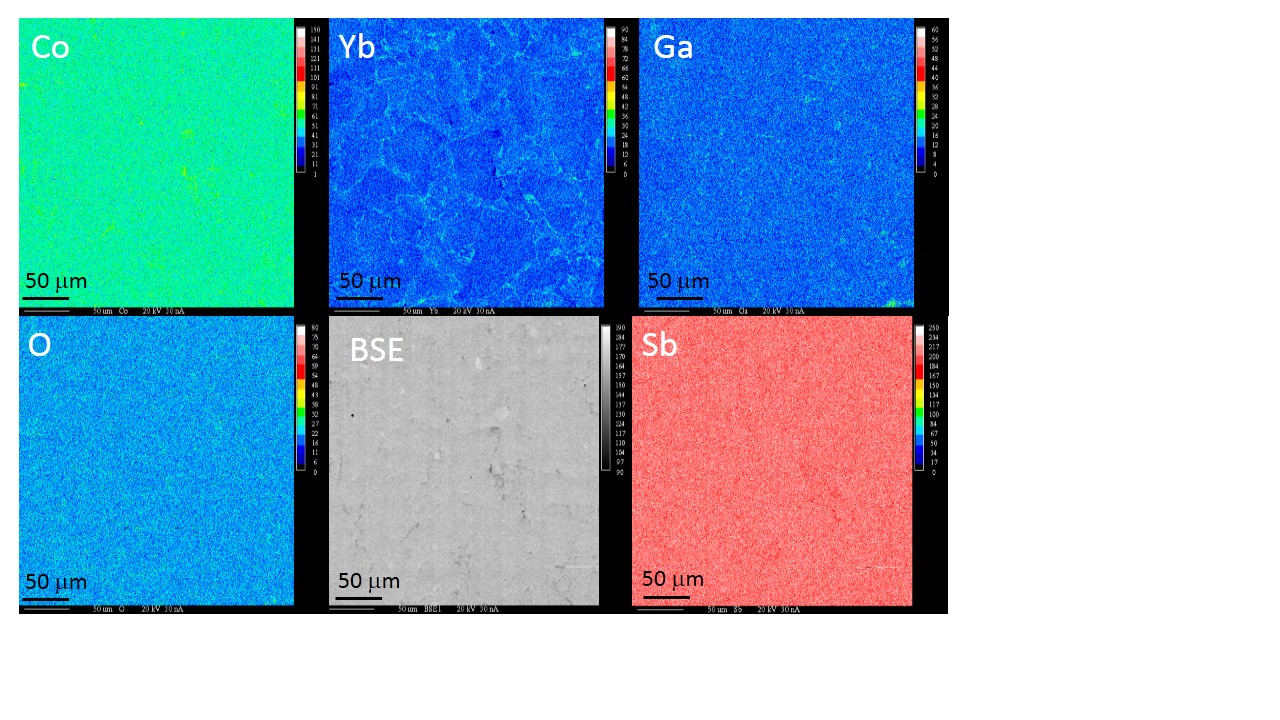


Supplementary Figure 1. EPMA mapping results of the sample Yb_0.30_Ga_0.15_Co_4_Sb_11.95_. With the exception of Yb the maps above are very uniform indicating a homogenous distribution of the elements with very little to no oxide content. EPMA also finds that all four constituent elements are present in each grain.


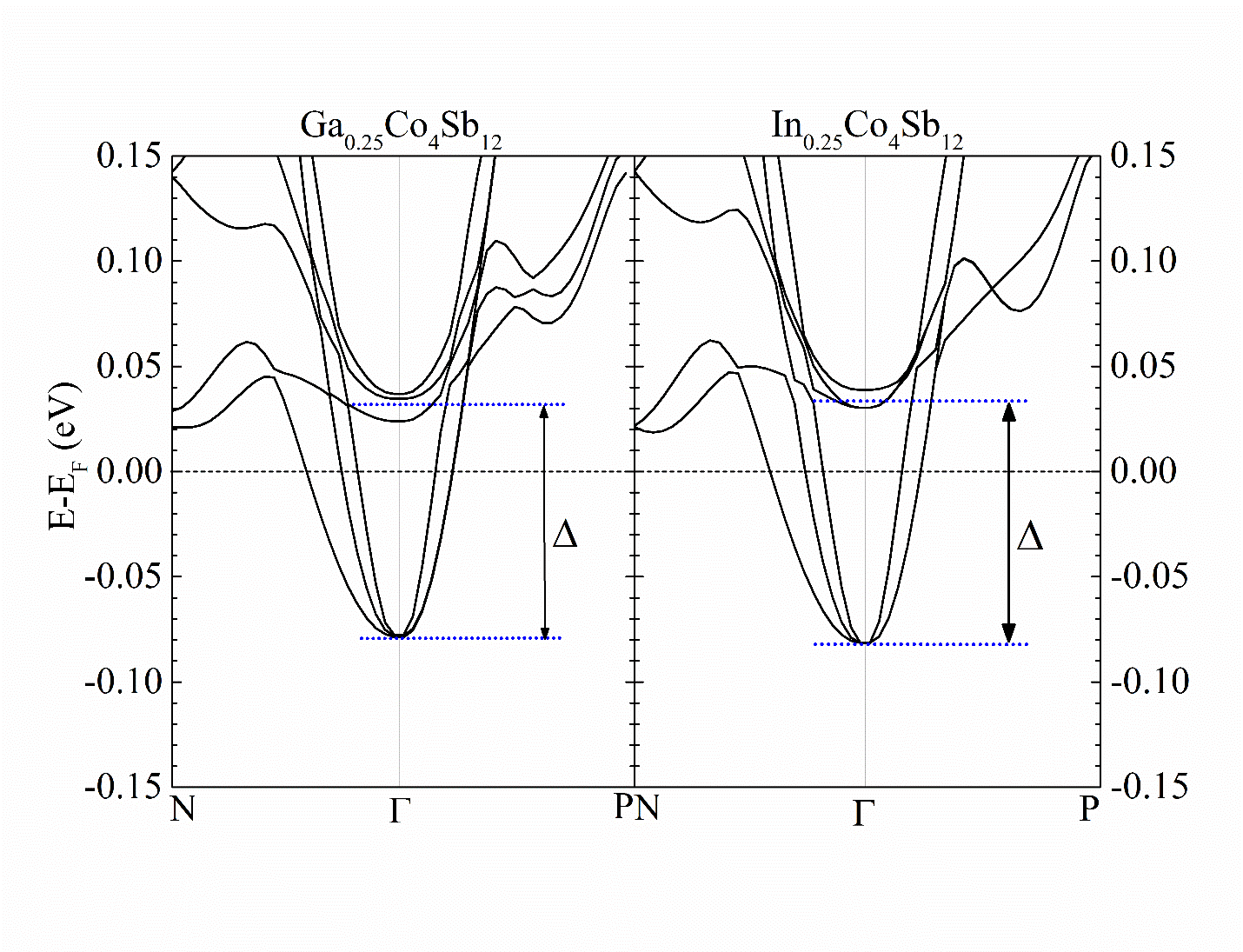


Supplementary Figure 2. First-principles band structure calculations. The left is Ga_0.25_Co_4_Sb_12_ and the right is In_0.25_Co_4_Sb_12_, assuming the IIIA atoms are all at filler sites. The zero energy points represent the Fermi levels (E_F_s) of the two compounds.
